# The triglyceride-glucose index predicts 1-year major adverse cardiovascular events in end-stage renal disease patients with coronary artery disease

**DOI:** 10.1186/s12933-023-02028-7

**Published:** 2023-10-27

**Authors:** Enmin Xie, Zixiang Ye, Yaxin Wu, Xuecheng Zhao, Yike Li, Nan Shen, Yanxiang Gao, Jingang Zheng

**Affiliations:** 1 Department of Cardiology, China-Japan Friendship Hospital, Beijing, China; 2 China-Japan Friendship Hospital (Institute of Clinical Medical Sciences), Chinese Academy of Medical Sciences, Peking Union Medical College, Beijing, China; 3 Department of Cardiology, Peking University China-Japan Friendship School of Clinical Medicine, Beijing, China; 4 Department of Cardiology, Henan Provincial People’s Hospital, Fuwai Central China Cardiovascular Hospital, Zhengzhou, China

**Keywords:** Triglyceride-glucose index, Insulin resistance, Coronary artery disease, End-stage renal disease, Major adverse cardiovascular events

## Abstract

**Background:**

The triglyceride-glucose (TyG) index has been suggested as a dependable indicator for predicting major adverse cardiovascular events (MACE) in individuals with cardiovascular conditions. Nevertheless, there is insufficient data on the predictive significance of the TyG index in end-stage renal disease (ESRD) patients with coronary artery disease (CAD).

**Methods:**

This study, conducted at multiple centers in China, included 959 patients diagnosed with dialysis and CAD from January 2015 to June 2021. Based on the TyG index, the participants were categorized into three distinct groups. The study’s primary endpoint was the combination of MACE occurring within one year of follow-up, including death from any cause, non-fatal myocardial infarction, and non-fatal stroke. We assessed the association between the TyG index and MACE using Cox proportional hazard models and restricted cubic spline analysis. The TyG index value was evaluated for prediction incrementally using C-statistics, continuous net reclassification improvement (NRI), and integrated discrimination improvement (IDI).

**Results:**

The three groups showed notable variations in the risk of MACE (16.3% in tertile 1, 23.5% in tertile 2, and 27.2% in tertile 3; log-rank *P* = 0.003). Following complete adjustment, patients with the highest TyG index exhibited a notably elevated risk of MACE in comparison to those in the lowest tertile (hazard ratio [HR] 1.63, 95% confidence interval [CI] 1.14–2.35, *P* = 0.007). Likewise, each unit increase in the TyG index correlated with a 1.37-fold higher risk of MACE (HR 1.37, 95% CI 1.13–1.66, *P* = 0.001). Restricted cubic spline analysis revealed a connection between the TyG index and MACE (*P* for nonlinearity > 0.05). Furthermore, incorporating the TyG index to the Global Registry of Acute Coronary Events risk score or baseline risk model with fully adjusted factors considerably enhanced the forecast of MACE, as demonstrated by the C-statistic, continuous NRI, and IDI.

**Conclusions:**

The TyG index might serve as a valuable and dependable indicator of MACE risk in individuals with dialysis and CAD, indicating its potential significance in enhancing risk categorization in clinical settings.

**Supplementary Information:**

The online version contains supplementary material available at 10.1186/s12933-023-02028-7.

## Background

Cardiovascular events are significantly more likely to occur in patients with end-stage renal disease (ESRD), who are considered a highly susceptible subset of coronary artery disease (CAD) [1, 2]. The risk of one-year major adverse cardiovascular events (MACE) for these patients is more than five times greater than that of patients without ESRD [3, 4]. Notably, the excess risk in patients with ESRD and CAD cannot be solely attributed to traditional cardiovascular risk factors [1, 5]. Hence, it is clinically significant for patients with ESRD and CAD to further investigate prognostic factors that indicate other aspects of the disease and discover potential treatment targets.

Insulin resistance (IR) is an eminent characteristic in ESRD patients [6–8]. The triglyceride-glucose (TyG) index calculated using fasting blood glucose (FBG) and triglyceride (TG), has become a dependable substitute indicator for IR [9]. Numerous studies have shown a robust correlation between the TyG index and conventional measures of IR, such as the hyperinsulinemic-euglycemic glucose clamp and the homeostasis model assessment for IR [10]. Multiple investigations have uncovered a favorable correlation between an elevated TyG index and the occurrence of heart disease [11], the severity of CAD [12], coronary artery calcification [13], and adverse cardiovascular events [14]. However, these studies have notably underrepresented or excluded patients with ESRD and CAD.

Notably, lipid and glucose metabolism undergo distinctive alterations with the decline in renal function [15, 16]. In the context of ESRD, especially among those requiring dialysis therapy, there is even a phenomenon known as 'reverse epidemiology'. In these patients, conventional cardiovascular risk factors often exhibit opposing effects as compared to the general population. For example, higher TG levels are associated with paradoxically lower mortality risk, while lower TG levels are linked to higher mortality risk [17, 18]. Similar trends are noted with total cholesterol (TC) and low-density lipoprotein cholesterol (LDL-C) [17, 19]. Furthermore, the kidney plays a central role in glucose homeostasis, and ESRD patients on dialysis frequently experience disrupted glucose and insulin regulation [20]. These changes in glucose and insulin homeostasis, along with modified responses to glucose-lowering therapies, increase susceptibility to both hypoglycemia and hyperglycemia [20, 21]. Given distinctive changes in lipid and glucose metabolism, it may not be appropriate to extrapolate findings from CAD patients with normal kidney function to ESRD patients on dialysis with CAD. However, the association between the TyG index and adverse outcomes remained unexplored in these patients. The unknown remains regarding the incremental predictive value of the TyG index for patients with ESRD and CAD. The objective of this study was to assess the predictive significance of the TyG index in estimating the risk of MACE within one year in Chinese patients with ESRD and CAD, utilizing data from a multi-center cohort investigation.

## Methods

### Study design

Data from the CRUISE-R study (ClinicalTrials.gov NCT05841082) were employed in this research, which focused on coronary revascularization in dialysis patients in China. The CRUISE-R study was a multi-center observational registry that aimed to examine the clinical features, healthcare, and predictive elements of patients with ESRD receiving dialysis and having CAD. The registry evaluated 455,617 cardiac catheterizations conducted between January 2015 and June 2021. Exclusion criteria were rigorously applied, which included patients who did not receive dialysis therapy or received dialysis therapy for less than 3 months (n = 453,421), individuals without any coronary stenosis exceeding 50% (n = 328), and patients with other indications for coronary angiography (n = 87). In the case of readmitted patients, only data from their initial admission were analyzed, while subsequent readmissions were documented as "readmission" events (n = 532). Consequently, a total of 1249 patients on dialysis with obstructive CAD were enrolled in the registry.

**Fig. 1:**
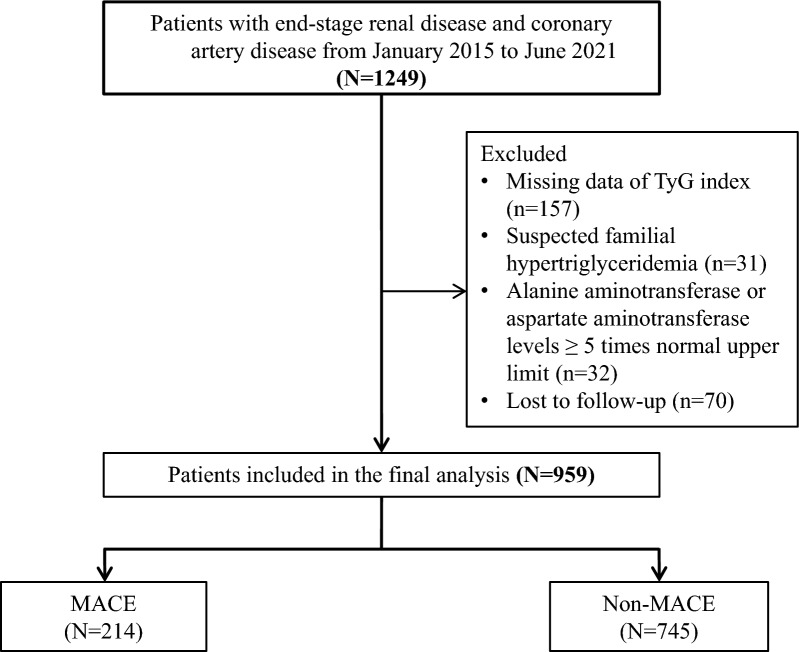
Flow diagram of patient’s selection. TyG, triglyceride-glucose index; MACE, major adverse cardiovascular events

For the present analysis, we further excluded 31 individuals who were suspected of having familial hypertriglyceridemia (TG ≥ 5.65 mmol/L), 157 patients lacking necessary data for TyG index calculation, as well as 32 patients who had alanine aminotransferase or aspartate aminotransferase levels that exceeded the normal upper limit by ≥ 5 times. Furthermore, a total of 70 patients who were lost to one-year follow-up were not included. In the end, a total of 959 participants were included in the present analysis (Fig. 1). The research followed the guidelines of the Strengthening the Reporting of Observational Studies in Epidemiology (STROBE) statement.

The research was carried out following the guidelines of the Declaration of Helsinki and obtained approval from the Ethics Committee of China-Japan Friendship Hospital (No. 2020-112-K71), with an exemption from the requirement of informed consent.

### Gathering of information and definitions

Data were collected from electronic medical records at each participating center by qualified study coordinators. The initial demographic and clinical data consisted of age, sex, blood pressure, heart rate, diabetes, active smokers, atrial fibrillation, cerebrovascular disorder, valve disorder, peripheral artery disease, primary presentation, dialysis method, length of dialysis (vintage), and reason for dialysis. Additionally, various laboratory tests were conducted to obtain measurements including hemoglobin, serum creatinine, TG, TC, LDL-C, and high-density lipoprotein cholesterol (HDL-C). The information regarding coronary angiography comprised the technique employed for access, the extent of disease, the existence of moderate or severe calcification, and the application of percutaneous coronary intervention (PCI) for treatment. In addition, documentation was made regarding the usage of medications, which included dual antiplatelet therapy, angiotensin-converting enzyme inhibitor or angiotensin receptor blocker, beta-blocker, calcium channel blocker, and statin. Survival and clinical assessment data were collected by trained nurses through outpatient clinic visits and telephone interviews.

Hypertension was defined by meeting any of the following criteria: systolic blood pressure equal to or greater than 140 mmHg, diastolic blood pressure equal to or greater than 90 mmHg, or the use of antihypertensive medication. Diabetes was defined by considering the following factors: the usage of oral medications for lowering blood sugar or insulin, or HbA1c levels equal to or greater than 6.5% upon admission. The formula used to calculate the TyG index is as follows: ln [(TG (mg/dl) × glucose (mg/dl)/2], using glucose and TG levels obtained within 24 h of admission [9]. Data required for calculating the Global Registry of Acute Coronary Events (GRACE) risk scores, ranging from 1 to 372, were derived from hospital admission records [22]. Variables considered during the calculation included age, heart rate, systolic blood pressure, creatinine levels, Killip class, occurrence of cardiac arrest during admission, presence of ST-segment deviation, and levels of cardiac biomarkers.

### Outcomes and follow-up

The study’s primary endpoint was the incidence of MACE during the one-year follow-up period, which included death from any cause, non-fatal myocardial infarction, and non-fatal stroke. Each individual component of MACE was recorded as a secondary endpoint. Furthermore, we also recorded gastrointestinal bleeding as a secondary endpoint, which was defined as hematemesis, coffee-ground emesis, melena, a significant drop in hemoglobin with a heme-positive stool test, or hematochezia as documented by the treating physician. The diagnosis of myocardial infarction was made by treating clinicians following a comprehensive evaluation that considered multiple factors. These factors included the existence of ischemic symptoms, increased levels of cardiac biomarkers in the blood, and/or noticeable changes in the electrocardiogram. Diagnosing a stroke involves identifying a fresh neurological impairment caused by vascular issues in the central nervous system, backed by imaging proof from computed tomography or magnetic resonance imaging. For patients experiencing multiple events, only the initial occurrence was considered for analysis.

### Statistical analysis

Patients were categorized into three groups based on the TyG index value. The mean and standard deviation or the median and interquartile range (25th, 75th percentile) are used to display continuous variables, and they are compared using the ANOVA test or the Kruskal–Wallis H test, as deemed appropriate. Categorical variables were analyzed by calculating frequencies and percentages, and then compared using either the Chi-square test or the Fisher exact test, depending on the circumstances. The Kaplan–Meier (KM) method was used to create cumulative curves for primary and secondary endpoints, and the log-rank test was utilized to differentiate between the curves of the groups. Univariable and multivariable Cox proportional hazard models were constructed to assess the correlation between the TyG index and clinical outcomes. Model 1 remained unchanged, whereas Model 2 included age and gender as factors. The Model 3 was completely calibrated. Table 1 contained the candidate variables, with Model 3 including confounders that were either statistically significant or clinically relevant. The hazard ratios (HRs) along with a corresponding 95% confidence interval (CI) were presented as the outcomes obtained from the Cox regression model. Proportional hazard assumptions were validated using Schoenfeld residuals. Multiple imputation techniques were applied to estimate missing data values. Moreover, an analysis using restricted cubic spline (RCS) was performed with four knots to identify any possible nonlinear associations between the TyG index and outcomes. Model 3 included confounding factors that were adjusted in the restricted cubic spline model. Since there is currently no specific risk score tailored to ESRD patients on dialysis with CAD, we assess the predictive value of the GRACE score in our study, considering its widespread use in the field. Moreover, we aimed to investigate whether the TyG index could enhance the predictive performance of the GRACE score in these patients. To assess the incremental predictive performance of outcomes after introducing the TyG index to the GRACE risk score or the baseline risk model with fully adjusted variables, various measures were used including the calculation of the C statistic, continuous net reclassification improvement (NRI), and integrated discrimination improvement (IDI). The C statistic was calculated to represent the performance of each model using “Survival” R package, while the incremental prognostic value of the TyG index on model fit was compared by likelihood ratio test. Both the continuous NRI and IDI were calculated using “survIDINRI” R package. Variables such as age, gender, diabetes mellitus, smoking, dialysis modality, index presentation, insulin therapy, and PCI treatment were taken into account during subgroup analysis. Moreover, a sensitivity analysis was performed to evaluate the strength of the main results by excluding patients who encountered a MACE occurrence while being hospitalized. Furthermore, considering the potential influence of hypoglycemia on the association between the TyG index and MACE, we conducted a sensitivity analysis by excluding individuals with blood glucose levels below 3.9 mmol/L. Given the observational nature of this study, we used propensity score matching (PSM) to reduce potential selection bias and balance baseline characteristics between groups. First, we performed receiver operating characteristic curve analysis to determine the optimal cut-off value of the TyG index for predicting the occurrence of MACE. The PSM analysis employs a 1:1 matching protocol without replacement, utilizing the greedy matching algorithm and a caliper width equivalent to 0.2 of the standard deviation of the propensity score logit. The estimation of the propensity score was through a multivariable logistic regression model, which included most of the covariates listed in Table 1, with the exclusion of glucose and triglycerides. Covariate balance between groups before and after PSM was assessed using mean absolute standardized differences, with differences less than 10% indicating good balance. Statistical analyses utilized two-sided P-values, with significance determined at a level below 0.05. The data was analyzed using SPSS 23.0 from IBM SPSS 23 Inc and R 3.6.1 by the R Development Core Team in Vienna, Austria.

**Table 1:** Baseline clinical characteristics of patients according to tertiles of triglyceride-glucose index

Characteristic	Total *N* = 959	Tertile 1, ≤ 8.77 *N* = 320	Tertile 2, 8.77–9.40 *N* = 319	Tertile 3, > 9.40 *N* = 320	*P* value
Age, mean (SD), years	61.8 (10.5)	62.3 (10.9)	60.8 (10.5)	62.4 (9.9)	0.080
Male, No. (%)	715 (74.6)	246 (76.9)	235 (73.7)	234 (73.1)	0.500
SBP, mean (SD), mmHg	141.6 (25.0)	142.6 (24.7)	142.6 (24.7)	139.6 (25.5)	0.206
DBP, mean (SD), mmHg	78.7 (13.4)	79.6 (13.4)	79.4 (13.6)	77.2 (13.1)	0.046
Heart rate, mean (SD), beats/min	80.5 (14.7)	78.8 (13.9)	81.0 (15.7)	81.7 (14.3)	0.034
Medical history and risk factors, No. (%)					
Hypertension	890 (92.8)	288 (90.0)	303 (95.0)	299 (93.4)	0.044
Diabetes mellitus	509 (53.1)	116 (36.2)	165 (51.7)	228 (71.2)	< 0.001
Current smoker	181 (18.9)	65 (20.3)	56 (17.6)	60 (18.8)	0.671
Atrial fibrillation	81 (8.4)	26 (8.1)	27 (8.5)	28 (8.8)	0.960
Cerebrovascular disease	184 (19.2)	61 (19.1)	59 (18.5)	64 (20.0)	0.888
Valvular disease	31 (3.2)	13 (4.1)	12 (3.8)	6 (1.9)	0.237
Peripheral arterial disease	95 (9.9)	29 (9.1)	29 (9.1)	37 (11.6)	0.478
Cause of ESRD, No. (%)					< 0.001
Diabetes mellitus	304 (31.7)	67 (20.9)	100 (31.3)	137 (42.8)	
Hypertension	123 (12.8)	55 (17.2)	39 (12.2)	29 (9.1)	
Glomerulonephritis	230 (24.0)	102 (31.9)	68 (21.3)	60 (18.8)	
Other/unknown	302 (31.5)	96 (30.0)	112 (35.1)	94 (29.4)	
Insulin therapy, No. (%)	322 (33.6)	65 (20.3)	102 (32.0)	155 (48.4)	< 0.001
Dialysis modality, No. (%)					0.065
Hemodialysis	887 (92.5)	300 (93.8)	300 (94.0)	287 (89.7)	
Peritoneal dialysis	72 (7.5)	20 (6.2)	19 (6.0)	33 (10.3)	
Vintage, years					0.624
< 1	199 (20.8)	69 (21.6)	62 (19.4)	68 (21.2)	
1–5	444 (46.3)	141 (44.1)	147 (46.1)	156 (48.8)	
5–10	248 (25.9)	83 (25.9)	91 (28.5)	74 (23.1)	
≥ 10	68 (7.1)	27 (8.4)	19 (6.0)	22 (6.9)	
Index presentation, No. (%)					0.292
AMI	564 (58.8)	177 (55.3)	192 (60.2)	195 (60.9)	
Non-AMI	395 (41.2)	143 (44.7)	127 (39.8)	125 (39.1)	
Hemoglobin, g/L	105.5 (19.8)	104.7 (19.0)	106.0 (19.2)	105.7 (21.2)	0.668
Glucose, mmol/L	6.4 [4.8, 9.3]	4.7 [4.2, 5.7]	6.3 [5.0, 8.0]	10.0 [7.3, 13.5]	< 0.001
Serum creatinine, mg/dl	8.7 [6.7, 10.9]	8.5 [6.9, 10.5]	9.2 [7.2, 11.7]	8.0 [6.2, 10.5]	0.001
TG, mmol/L	1.6 [1.1, 2.3]	1.0 [0.8, 1.3]	1.7 [1.4, 2.1]	2.5 [1.9, 3.4]	< 0.001
TC, mmol/L	3.7 [3.1, 4.5]	3.5 [2.9, 4.1]	3.6 [3.2, 4.4]	4.1 [3.2, 4.9]	< 0.001
HDL-C, mmol/L	0.9 [0.7, 1.1]	1.0 [0.8, 1.2]	0.8 [0.7, 1.1]	0.8 [0.7, 1.0]	< 0.001
LDL-C, mmol/L	2.2 [1.7, 2.8]	2.1 [1.6, 2.5]	2.2 [1.7, 2.8]	2.3 [1.7, 3.0]	0.001
TyG index	9.1 [8.6, 9.6]	8.3 [8.1, 8.6]	9.1 [8.9, 9.2]	9.8 [9.6, 10.1]	< 0.001
Procedure characteristic, No. (%)					
Radial access	740 (77.2)	241 (75.3)	249 (78.1)	250 (78.1)	0.627
Extent of disease					
Any left main disease	111 (11.6)	32 (10.0)	44 (13.8)	35 (10.9)	0.296
2-vessel disease	263 (27.4)	85 (26.6)	82 (25.7)	96 (30.0)	0.436
3-vessel disease	544 (56.7)	169 (52.8)	184 (57.7)	191 (59.7)	0.196
Moderate or severe calcification	432 (45.0)	141 (44.1)	149 (46.7)	142 (44.4)	0.764
PCI treatment	676 (70.5)	218 (68.1)	225 (70.5)	233 (72.8)	0.429
Discharge medications, No. (%)					
Dual antiplatelet therapy	836 (87.2)	277 (86.6)	286 (89.7)	273 (85.3)	0.240
ACE inhibitor or ARB	441 (46.0)	160 (50.0)	142 (44.5)	139 (43.4)	0.203
Beta-blocker	775 (80.8)	248 (77.5)	261 (81.8)	266 (83.1)	0.167
Calcium-channel blocker	620 (64.7)	220 (68.8)	206 (64.6)	194 (60.6)	0.099
Statin	899 (93.7)	296 (92.5)	302 (94.7)	301 (94.1)	0.505

Data are presented as mean (SD) or n (%)

ACE, angiotensin-converting enzyme; AMI, acute myocardial infarction; ARB, angiotensin receptor blocker; DBP, diastolic blood pressure; ESRD, end-stage renal disease; HDL-C, high-density lipoprotein cholesterol; LDL-C, low-density lipoprotein cholesterol; PCI, percutaneous coronary intervention; SBP, systolic blood pressure; TC, total cholesterol; TG, triglycerides; TyG, triglyceride-glucose

## Results

### Baseline characteristics

Overall, 715 (74.6%) patients were men (61.8 ± 10.5 years). Hypertension (92.8%) was the most common comorbidity. The median TyG index for the entire group was 9.1, with an interquartile range spanning from 8.6 to 9.6. Table 1 displays the demographic and clinical features of the three groups classified based on the TyG index. Individuals with elevated TyG index demonstrated an increased occurrence of diabetes, insulin therapy, and diabetes as the primary factor leading to ESRD. Furthermore, individuals in the third tertile of the TyG index exhibited an elevated heart rate, TC, and LDL-C, while experiencing a reduced diastolic blood pressure, serum creatinine, and HDL-C. During an 835 person-year follow-up, a cumulative of 214 (22.3%) MACE incidents were documented, which comprised 156 (16.3%) cases of all-cause mortality, 53 (5.5%) non-fatal myocardial infarction, and 12 (1.3%) non-fatal stroke. Furthermore, 38 (4.0%) gastrointestinal bleedings were recorded, with 20 resulting in hospitalization and 2 resulting in death. The comparison of baseline characteristics between patients with and without MACE was shown in Table 2. Typically, individuals with a higher occurrence of diabetes, atrial fibrillation, acute myocardial infarction as the initial manifestation, left main disease, and 3-vessel disease were the ones who encountered MACE. In contrast, the percentage of individuals receiving angiotensin-converting enzyme inhibitor or angiotensin receptor blocker therapy and calcium-channel blocker therapy was lower. Furthermore, patients with diabetes demonstrated significantly higher TyG index values than those without diabetes mellitus (9.3 [8.8–9.8] vs 8.8 [8.3–9.3], *P* < 0.001). Furthermore, among patients with diabetes, those who experienced MACE exhibited significantly higher TyG index values compared to those without MACE (9.4 [9.1, 9.9] vs 9.3 [8.8, 9.7], *P* = 0.018). This pattern was similarly observed in patients without diabetes (9.0 [8.4, 9.4] vs 8.8 [8.3, 9.2], *P* = 0.019).

**Table 2:** Baseline clinical characteristics of patients stratified by major adverse cardiovascular events

Characteristic	Non-MACE (n = 745)	MACE (n = 214)	*P* value
Age, mean (SD), years	61.2 (10.6)	63.9 (9.9)	0.001
Male, No. (%)	556 (74.6)	159 (74.3)	0.993
SBP, mean (SD), mmHg	143.5 (25.0)	134.9 (23.9)	< 0.001
DBP, mean (SD), mmHg	79.8 (13.5)	74.9 (12.5)	< 0.001
Heart rate, mean (SD), beats/min	79.9 (14.4)	82.4 (15.6)	0.026
Medical history and risk factors, No. (%)			
Hypertension	689 (92.5)	201 (93.9)	0.569
Diabetes mellitus	380 (51.0)	129 (60.3)	0.020
Current smoker	142 (19.1)	39 (18.2)	0.860
Atrial fibrillation	55 (7.4)	26 (12.1)	0.038
Cerebrovascular disease	133 (17.9)	51 (23.8)	0.063
Valvular disease	22 (3.0)	9 (4.2)	0.488
Peripheral arterial disease	70 (9.4)	25 (11.7)	0.391
Cause of ESRD, No. (%)			0.077
Diabetes mellitus	226 (30.3)	78 (36.4)	
Hypertension	91 (12.2)	32 (15.0)	
Glomerulonephritis	191 (25.6)	39 (18.2)	
Other/unknown	237 (31.8)	65 (30.4)	
Insulin therapy, No. (%)	246 (33.0)	76 (35.5)	0.549
Dialysis modality, No. (%)			1.000
Hemodialysis	689 (92.5)	198 (92.5)	
Peritoneal dialysis	56 (7.5)	16 (7.5)	
Vintage, years			0.331
< 1	162 (21.7)	37 (17.3)	
1–5	337 (45.2)	107 (50.0)	
5–10	190 (25.5)	58 (27.1)	
≥ 10	56 (7.5)	12 (5.6)	
Index presentation, No. (%)			< 0.001
AMI	413 (55.4)	151 (70.6)	
Non-AMI	332 (44.6)	63 (29.4)	
Hemoglobin, g/L	105.3 (19.9)	105.9 (19.5)	0.696
Glucose, mmol/L	6.0 [4.7, 8.7]	7.9 [5.7, 10.6]	< 0.001
Serum creatinine, mg/dl	8.7 [6.7, 10.9]	8.4 [6.7, 10.9]	0.416
TG, mmol/L	1.6 [1.1, 2.3]	1.6 [1.1, 2.3]	0.585
TC, mmol/L	3.7 [3.1, 4.5]	3.8 [3.1, 4.4]	0.854
HDL-C, mmol/L	0.9 [0.7, 1.1]	0.9 [0.7, 1.1]	0.204
LDL-C, mmol/L	2.2 [1.6, 2.8]	2.1 [1.7, 2.8]	0.894
TyG index	9.0 [8.5, 9.5]	9.3 [8.8, 9.8]	< 0.001
Procedure characteristic, No. (%)			
Radial access	578 (77.6)	162 (75.7)	0.627
Extent of disease			
Any left main disease	67 (9.0)	44 (20.6)	< 0.001
2-vessel disease	216 (29.0)	47 (22.0)	0.052
3-vessel disease	391 (52.5)	153 (71.5)	< 0.001
Moderate or severe calcification	324 (43.5)	108 (50.5)	0.084
PCI treatment	535 (71.8)	141 (65.9)	0.112
Discharge medications, No. (%)			
Dual antiplatelet therapy	642 (86.2)	194 (90.7)	0.107
ACE inhibitor or ARB	357 (47.9)	84 (39.3)	0.030
Beta-blocker	597 (80.1)	178 (83.2)	0.369
Calcium-channel blocker	500 (67.1)	120 (56.1)	0.004
Statin	697 (93.6)	202 (94.4)	0.776

Data are presented as mean (SD) or n (%)

ACE, angiotensin-converting enzyme; AMI, acute myocardial infarction; ARB, angiotensin receptor blocker; DBP, diastolic blood pressure; ESRD, end-stage renal disease; HDL-C, high-density lipoprotein cholesterol; LDL-C, low-density lipoprotein cholesterol; PCI, percutaneous coronary intervention; SBP, systolic blood pressure; TC, total cholesterol; TG, triglycerides; TyG, triglyceride-glucose

### The correlation between the TyG index and MACE

**Fig. 2:**
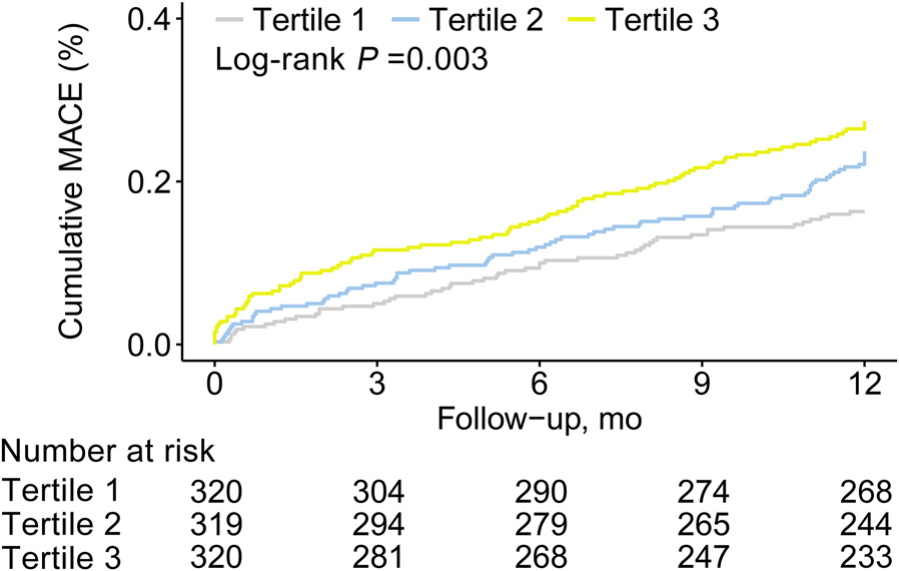
Kaplan–Meier Estimated Event Rates of major adverse cardiovascular events according to tertiles of the triglyceride-glucose index. MACE, major adverse cardiovascular events

**Fig. 3:**
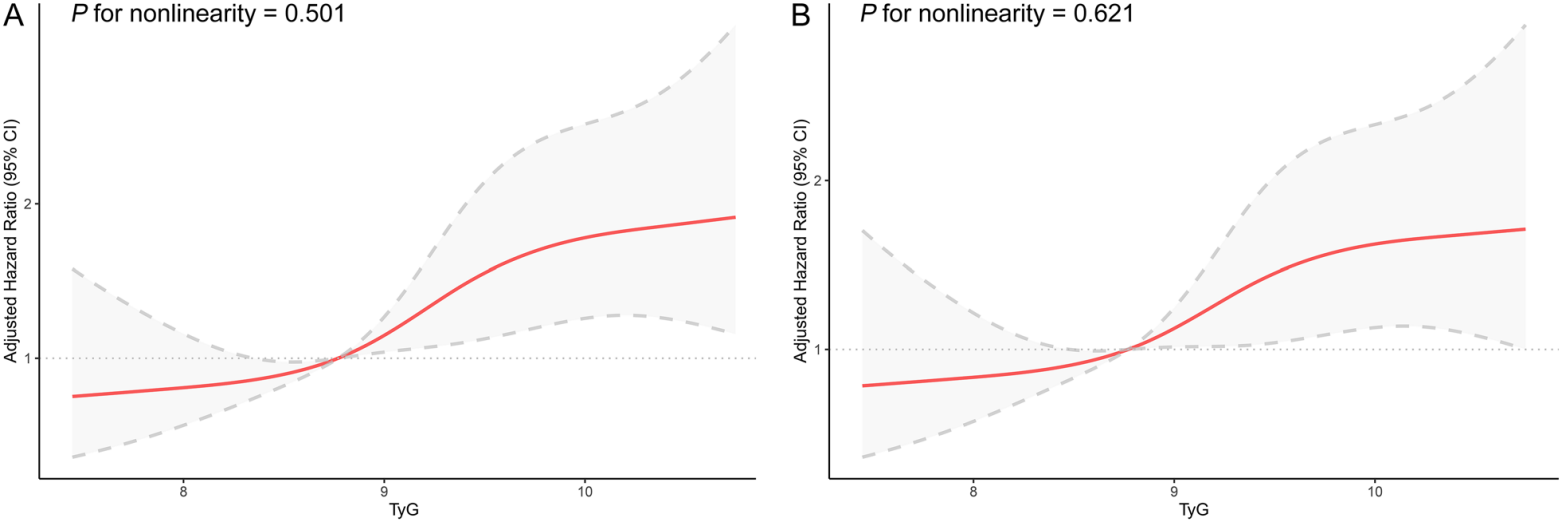
Restricted cubic spline curves of the association between triglyceride-glucose index and major adverse cardiovascular events. **A** Unadjusted model. **B** fully adjusted model. Hazard ratios are indicated by solid red lines and 95% CIs are indicated by shaded areas. CI, confidence interval; TyG, triglyceride-glucose index

As the tertile of the TyG index increased, the occurrence of MACE showed a gradual rise with percentages of 16.3% (52/320), 23.5% (75/319), and 27.2% (87/320), respectively. In Fig. 2, it can be observed that KM curves showed a noticeably increased risk of MACE in patients belonging to the third TyG tertiles when compared to the remaining groups (log-rank test *P* = 0.003). Table 3 presents the correlation between the TyG index and MACE. The TyG index showed a significant positive correlation with MACE (HR, 1.44; 95% CI, 1.21–1.72) according to the univariate Cox regression analysis (Model 1). After accounting for age and gender, we observed a similar positive correlation (Model 2). After making comprehensive adjustments for initial clinical risk factors, there was a notable increase in the risk of MACE associated with a rising TyG index (adjusted hazard ratio, 1.37; 95% confidence interval, 1.13–1.66; *P* = 0.001) (Model 3). In the fully adjusted model, there was a notable rise in the risk of MACE when comparing the third TyG index tertile to the first TyG index tertile (adjusted HR, 1.63; 95% CI, 1.14–2.35; *P* = 0.007). Moreover, the analysis of RCS revealed a correlation between the TyG index and the risk of MACE, regardless of whether it was adjusted for baseline clinical risk factors in Model 3 or not (both *P* values for nonlinearity > 0.05) (refer to Fig. 3).

**Table 3:** Cox regression models for the association of triglyceride-glucose index and major adverse cardiovascular events

	Model 1			Model 2			Model 3		
	HR	95%CI	*P* value	HR	95%CI	*P* value	HR	95%CI	*P* value
TyG index	1.44	1.21–1.72	< 0.001	1.45	1.22–1.73	< 0.001	1.37	1.13–1.66	0.001
Tertile 1	Ref			Ref			Ref		
Tertile 2	1.49	1.05–2.13	0.027	1.57	1.10–2.23	0.013	1.45	1.01–2.09	0.047
Tertile 3	1.80	1.28–2.54	< 0.001	1.81	1.28–2.55	< 0.001	1.63	1.14–2.35	0.007

Model 1: unadjusted. Model 2: adjusted for age and sex. Model 3: adjusted for age, sex, systolic blood pressure, diastolic blood pressure, heart rate, diabetes mellitus, atrial fibrillation, cerebrovascular disease, cause of dialysis, acute myocardial infarction as index presentation, left main disease, 3-vessel disease, angiotensin-converting enzyme inhibitor or angiotensin receptor blocker, and calcium-channel blocker

CI, confidence interval; HR, hazard ratio; TyG, triglyceride-glucose

**Fig. 4:**
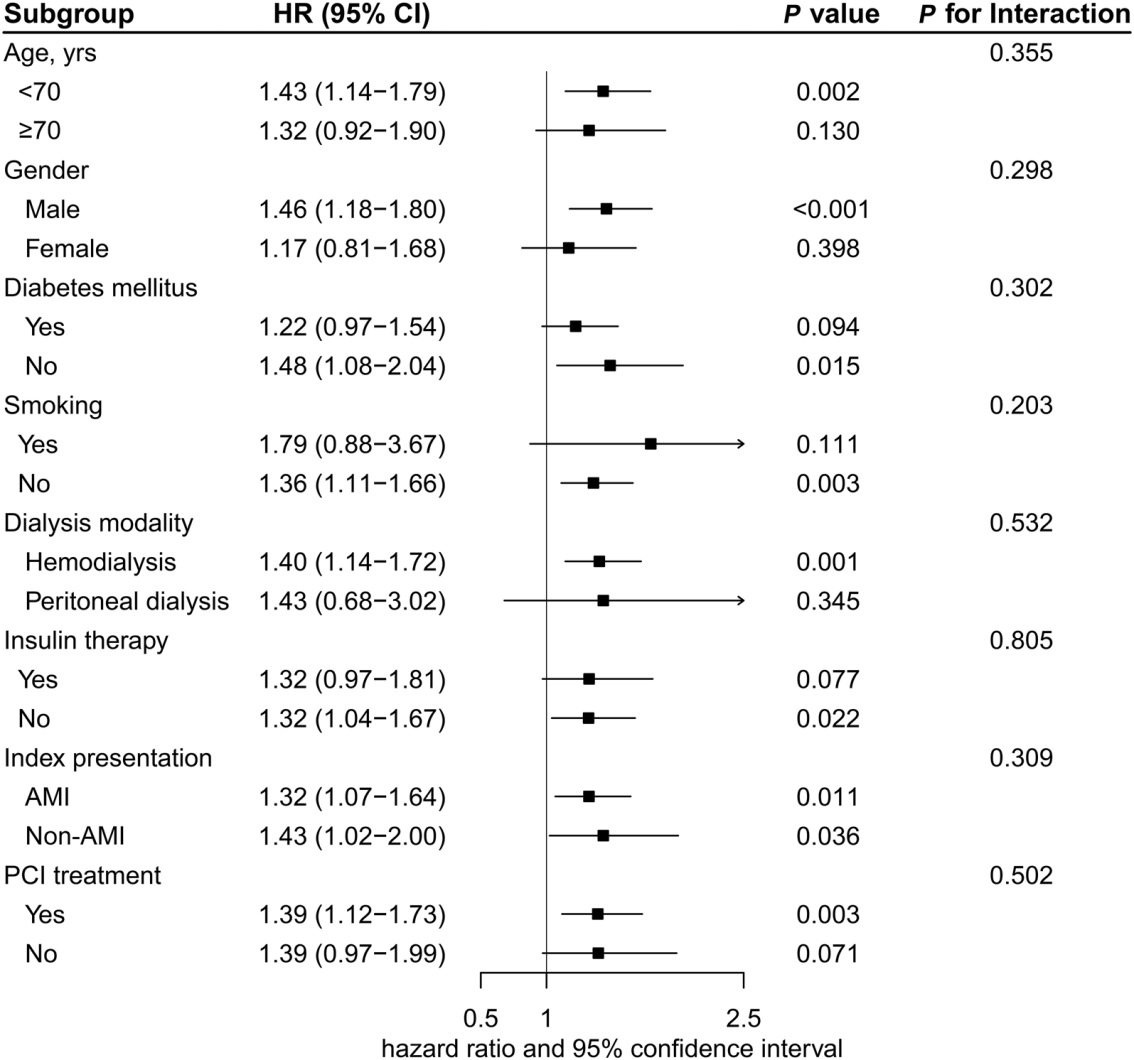
Subgroup Analyses for major adverse cardiovascular events. AMI, acute myocardial infarction; CI, confidence interval; HR, hazard ratio; PCI, percutaneous coronary intervention

Patients in subgroup analyses were categorized based on age, sex, diabetes, smoking, dialysis method, initial presentation, insulin therapy, and PCI intervention (with the TyG index considered as a continuous factor). No significant associations were found in the selected subgroups concerning the risk of MACE (all *P* values for interaction > 0.05) (Fig. 4).

Additional file 1: Table S1 presented the correlation between the TyG index and secondary outcomes. The third TyG index tertile in univariate Cox analysis was linked to a higher risk of all-cause mortality and non-fatal myocardial infarction, while no association was found with non-fatal stroke and gastrointestinal bleeding. The third TyG index tertile in the multivariable Cox regression model was found to be independently associated with all-cause death, but no statistically significant association was observed in predicting non-fatal myocardial infarction, stroke, and gastrointestinal bleeding.

### TyG index’s incremental predictive value

The predictive value of the TyG index for MACE was shown to increase in Table 4. By including the TyG index in the GRACE risk score, the ability to predict MACE improved, as evidenced by the C-statistic increasing from 0.634 to 0.653. As assessed by the likelihood ratio test, the model fit was improved with the addition of the TyG index (*P* < 0.001). We additionally assessed these enhancements utilizing the NRI, which produced a score of 0.129 (95% confidence interval, 0.041–0.203, *P* < 0.001), and the IDI, which yielded a score of 0.011 (95% confidence interval 0.001–0.030, *P* = 0.027). In addition, we removed the TyG index from Model 3 and conducted a baseline risk model to predict MACE (Additional file 1: Table S2). The Harrell’s C-indexes of the baseline risk model was 0.698. When incorporating the TyG index into the baseline risk model, a marginal improvement in the predictive ability for MACE was noted, as evidenced by the C-statistic rising from 0.698 to 0.705 (likelihood ratio test, *P* = 0.001). Significant enhancements in the NRI and IDI were also observed as a result of incorporating the TyG index into the initial risk model.

**Table 4:** Added predictive ability and reclassification statistics of triglyceride-glucose index

	C-statistic (95% CI)	*P *value	Continuous NRI (95% CI)	*P* value	IDI (95% CI)	*P* value
GRACE risk score	0.634 (0.597, 0.671)	< 0.001	Ref		Ref	
GRACE risk score + TyG index	0.653 (0.618, 0.688)	< 0.001	0.129 (0.041, 0.203)	< 0.001	0.011 (0.001, 0.030)	0.027
Baseline risk model^a^	0.698 (0.665, 0.731)	< 0.001	Ref		Ref	
Baseline risk model + TyG index	0.705 (0.672, 0.738)	< 0.001	0.093 (0.003, 0.185)	0.040	0.011 (0.001, 0.028)	0.020

CI, confidence interval; GRACE, Global Registry of Acute Coronary Events; IDI, integrated discrimination improvement; NRI, net reclassification improvement; TyG, triglyceride-glucose

^a^ Variables included in the baseline risk model for major adverse cardiovascular events are shown in Additional file 1: Table S2

### Sensitivity analysis

To evaluate the reliability of our primary findings, a sensitivity analysis was performed, excluding 41 patients who encountered a MACE incident while hospitalized. The KM curves demonstrated a significantly higher risk of MACE in patients belonging to the third TyG tertile group when compared to the remaining groups (Additional file 1: Figure S1). Multivariable Cox regression revealed a notable correlation between an elevated TyG index and an augmented risk of MACE, regardless of whether the TyG index was modeled as a continuous or categorical variable (Additional file 1: Table S3). In addition, the analysis using restricted cubic splines demonstrated a correlation between the TyG index and the risk of MACE, in both unadjusted and fully adjusted models (Additional file 1: Figure S2). Furthermore, after excluding 58 (6.0%) patients with blood glucose levels below 3.9 mmol/L, we still identified a significant association between the TyG index and MACE (Additional file 1: Table S4).

The receiver operating characteristic curve analysis was used to determine the optimal cut-off value of the TyG index for predicting MACE. This analysis identified an optimal cut-off value of 8.99 (sensitivity 69.16% and specificity 48.32%), with an area under the curve of 0.591 (95% confidence interval 0.548–0.634). The baseline and clinical characteristics of both the low TyG index (≤ 8.99) and high TyG index (> 8.99) groups are presented in Additional file 1: Table S5. Substantial differences were observed across several baseline characteristics before matching. Following the PSM analysis, a total of 265 pairs of patients in the low TyG index group and high TyG index group were successfully matched, resulting in cohorts with highly similar baseline characteristics (standardized differences < 0.10) (Additional file 1: Table S5). In the PSM cohort, the high TyG index remained significantly associated with an increased risk of MACE and all-cause death, while the risk of other secondary outcomes remained comparable (Table 5).

**Table 5:** Prognostic impact of triglyceride-glucose index after propensity score matching

Outcome	No. (%)		Unadjusted		Multivariable Adjusted	
	≤ 8.99	> 8.99	HR (95% CI)	*P* value	HR (95% CI)	*P* value
MACE	44 (16.6)	67 (25.3)	1.60 (1.10–2.34)	0.015	1.70 (1.15–2.50)	0.007
All-cause death	33 (12.5)	49 (18.5)	1.52 (0.98–2.37)	0.061	1.66 (1.06–2.59)	0.027
Non-fatal myocardial infarction	8 (3.0)	16 (6.0)	2.10 (0.90–4.91)	0.086	2.05 (0.88–4.81)	0.097
Non-fatal stroke	3 (1.1)	5 (1.9)	1.72 (0.41–7.21)	0.456	1.53 (0.36–6.47)	0.564
Gastrointestinal bleeding	11 (4.2)	14 (5.3)	1.30 (0.59–2.86)	0.516	1.24 (0.56–2.73)	0.599

CI, confidence interval; HR, hazard ratio; MACE, major adverse cardiovascular events

## Discussion

The predictive accuracy and clinical usefulness of the TyG index in patients with ESRD and CAD were assessed in this study conducted at a multi-center cohort study. According to our research, a greater TyG index was found to correlate with an elevated risk of MACE, displaying a noticeable pattern of increased risk with higher values. Furthermore, the integration of the TyG index into either the GRACE risk score or the baseline risk prediction model considerably enhanced the accuracy in predicting the risk of MACE. The findings emphasized the considerable clinical significance of a simple technique for assessing IR in the categorization of individuals with ESRD and CAD.

Due to its convenience, cost-effectiveness, and adaptability, the TyG index has gained significant popularity in medical settings as a reliable indicator for accurately assessingIR, demonstrating both high sensitivity and specificity [23]. Multiple research studies have shown that the TyG index has the capability to forecast negative cardiovascular results in different clinical presentations of CAD. According to a single-center retrospective cohort study, the TyG index independently increased the risk of MACE in individuals with premature CAD, regardless of traditional cardiovascular risk factors [24]. Likewise, a nested case–control investigation that concentrated on individuals with stable CAD revealed a favorable association between the TyG indicator and subsequent occurrences of cardiovascular events [25]. Furthermore, a research study including 639 individuals suffering from chronic kidney disease and CAD discovered that the TyG index can serve as a prognostic indicator for mortality within one year and during hospitalization [26]. Nevertheless, prior studies often overlooked or did not adequately include patients with ESRD, resulting in insufficient exploration of the prognostic potential of the TyG index in patients with both ESRD and CAD. According to projections, the quantity of individuals with ESRD is expected to increase twofold within the upcoming decade, with Asia witnessing the most significant surge in growth rates [27]. Cardiovascular disease is the main reason for illness and death in this group of patients, and CAD plays a significant role [1, 27]. Given distinctive changes in lipid and glucose metabolism, it may not be appropriate to extrapolate findings from CAD patients with normal kidney function to patients with ESRD and CAD [3, 4, 17–21]. Assessing the importance of the TyG index in risk stratification in this group of patients is of great therapeutic significance, considering the increasing number of individuals with ESRD and CAD, as well as the potential prognostic significance of the TyG index.

In this research, we discovered a correlation between the TyG and the risk of MACE after accounting for conventional risk factors in patients with ESRD and CAD. By employing RCS analysis, we noticed a pattern of response to varying doses. The findings of this study partially coincide with prior research, providing further evidence of the correlation between the TyG index and unfavorable prognostic outcomes in different clinical presentations of CAD [24–26]. Of note, the observed significant difference between the TyG index tertiles for MACE appeared to be primarily driven by an increase in all-cause mortality rather than other secondary outcomes. These findings align with certain aspects of previous research [24, 28] suggesting that the TyG index may function as an independent predictor of MACE rather than individual components of MACE. Given the relatively small number of secondary outcomes in our study, further research is warranted to confirm our findings. The connection between the TyG index and MACE is still not fully comprehended. Impaired renal function is a notable attribute in individuals suffering from ESRD [6–8]. The causes of this condition in these patients are diverse and intricate, encompassing uremic toxins, low red blood cell count, oxidative stress, persistent inflammation, metabolic acidosis, and an imbalanced gut microbiome [29–31]. Earlier research has demonstrated the role of IR in the development of atherosclerosis and subsequent negative cardiovascular events. These events may be caused by various molecular mechanisms such as endothelial dysfunction, coagulation abnormalities, impaired metabolic flexibility, and dysfunction of smooth muscle cells [32–37]. As a reliable indicator, the TyG index can effectively anticipate the likelihood of MACE in individuals diagnosed with ESRD and CAD. Further inquiry is necessary to fully understand the mechanisms that underpin the correlation between the TyG index and MACE in this group of patients.

In subgroup analysis, we observed an independent association between the TyG index and MACE in male patients, while no such association was found in female patients, with no significant interaction observed. These findings align with previous research to some extent, where a significant association between the TyG index and adverse cardiovascular events appeared to be more pronounced in male patients compared to female patients [38, 39]. Conversely, other studies have identified an independent association between the TyG index and poor prognosis in female patients rather than male patients [24, 40]. It is worth noting that the interaction tests in these studies did not reach statistical significance, consistent with our findings. These results collectively suggest that sex may not substantially modify the association between the TyG index and adverse events. Furthermore, we found that the TyG index was significantly higher in individuals with MACE compared to those without MACE, regardless of diabetes status. In subgroup analyses, we identified an independent association between the TyG index and MACE in patients without diabetes, in contrast to patients with diabetes, with no significant interaction observed. These findings are consistent with previous studies [23, 24] and indicate that the prognostic value of the TyG index was independent of diabetes mellitus.

An important discovery in this research was the substantial enhancement in forecast precision for MACE when integrating the TyG index into either the GRACE score or the baseline risk model with fully adjusted factors. The GRACE index, extensively employed in medical settings, has shown its efficacy in forecasting adverse cardiovascular results in patients with CAD [22, 41, 42]. Nonetheless, the researchers of the GRACE registry have recognized the inclination to underestimate the likelihood of unfavorable occurrences in individuals with ESRD undergoing dialysis [43]. The acknowledgment led to a deeper examination of including extra predictive variables in the GRACE score, intending to attain a more accurate assessment of prognosis and enhanced categorization of risk in this specific group of patients. The long-term prediction of MACE was enhanced by adding the TyG index to the GRACE score in a retrospective cohort study of 986 patients with acute coronary syndrome who underwent PCI [44]. Likewise, a separate investigation examined individuals with early-onset CAD and found that combining established risk factors with the TyG index improved the ability to classify the risk of severe cardiovascular events [24]. The focus of our research was specifically on individuals diagnosed with ESRD and CAD, offering novel perspectives on the predictive significance of the TyG index concerning patient outcomes. The results emphasize the significant significance of the TyG index in categorizing risk among individuals with ESRD and CAD. Ensuring a focused prognosis follow-up at an early stage is crucial for optimizing therapeutic regimens, and offering substantial clinical value. Additionally, more specific studies are necessary to examine if interventions that aim to address IR, as evaluated by the TyG index, can improve the clinical outlook for patients with ESRD and CAD.

This research examines the correlation between IR, as evaluated by the TyG indicator, and MACE in patients with ESRD and CAD. Nevertheless, our study has certain constraints. The study's retrospective nature gives rise to concerns regarding potential confounding factors and selection bias, which could influence the results. Furthermore, information regarding other glucose-related factors like diabetic drugs, history of severe hypoglycemia, and levels of insulin was not available. The calculation of the TyG index was derived from glucose and TG levels measured within 24 h of admission, potentially influenced by the fasting status. Moreover, there were no repeated assessments of the TyG, and the duration of the follow-up period was relatively short. While there is no standardized definition for myocardial infarction in dialysis patients, our study's significant association between the TyG index and MACE appears primarily influenced by all-cause death, making the specific definition of myocardial infarction less impactful. To address these constraints, it will be essential for future research to include larger sample sizes and more extensive data to confirm our findings and enhance our comprehension of these connections.

## Conclusions

In patients with ESRD and CAD, the TyG index could potentially serve as a reliable predictor of MACE. Incorporating the TyG index into the GRACE risk score or baseline risk model provided additional predictive value for forecasting MACE. Additional validation trials are necessary to confirm our findings and assess the potential benefits of therapies targeting IR in individuals with ESRD and CAD.

### Supplementary Information

**Additional file 1: Table S1.** Cox regression models for the association of triglyceride-glucose index and secondary outcomes. **Table S2.** Baseline risk model for major adverse cardiovascular events. **Table S3.** Cox regression models for the association of triglyceride-glucose index and MACE after excluding patients who experienced a MACE event during hospitalization. **Table S4.** Cox regression models for the association of triglyceride-glucose index and MACE after excluding patients with blood glucose levels below 3.9 mmol/L. **Table S5.** Baseline clinical characteristics of patients before and after propensity score matching. **Figure S1.** Kaplan-Meier Estimated Event Rates of MACE according to tertiles of the TyG index after excluding patients who experienced a MACE event during hospitalization. **Figure S2.** Restricted cubic spline curves of the association between TyG index and MACE after excluding patients who experienced a MACE event during hospitalization.

## Data Availability

The dataset analyzed during the current study is available from the corresponding author upon reasonable request.

## Abbreviations

CAD: Coronary artery disease
CI: Confidence interval
ESRD: End-stage renal disease
FPG: Fasting plasma glucose
GRACE: Global Registry of Acute Coronary Events
HDL-C: High-density lipoprotein cholesterol
IR: Insulin resistance
LDL-C: Low-density lipoprotein cholesterol
OR: Odds ratio
PCI: Percutaneous coronary intervention
TC: Total cholesterol
TG: Triglyceride
TyG: Triglyceride-glucose
MACE: Major adverse cardiovascular events
RCS: Restricted cubic spline
KM: Kaplan–Meier

## References

[1] Lai AC, Bienstock SW, Sharma R, Skorecki K, Beerkens F, Samtani R (2021). A personalized approach to chronic kidney disease and cardiovascular disease: JACC review topic of the week. J Am Coll Cardiol.

[2] Bello AK, Okpechi IG, Osman MA, Cho Y, Htay H, Jha V (2022). Epidemiology of haemodialysis outcomes. Nat Rev Nephrol.

[3] Limpijankit T, Chandavimol M, Srimahachota S, Kanoksilp A, Jianmongkol P, Siriyotha S (2022). Dose-dependent effect of impaired renal function on all-cause mortality in patients following percutaneous coronary intervention. Clin Cardiol.

[4] Tobe A, Sawano M, Kohsaka S, Ishii H, Tanaka A, Numasawa Y (2023). Ischemic and bleeding outcomes in patients who underwent percutaneous coronary intervention with chronic kidney disease or dialysis (from a Japanese Nationwide Registry). Am J Cardiol.

[5] Wanner C, Amann K, Shoji T (2016). The heart and vascular system in dialysis. Lancet.

[6] Krediet RT, Balafa O (2010). Cardiovascular risk in the peritoneal dialysis patient. Nat Rev Nephrol.

[7] Bernardo AP, Oliveira JC, Santos O, Carvalho MJ, Cabrita A, Rodrigues A (2015). Insulin resistance in nondiabetic peritoneal dialysis patients: associations with body composition, peritoneal transport, and peritoneal glucose absorption. Clin J Am Soc Nephrol.

[8] Nishimura M, Tsukamoto K, Tamaki N, Kikuchi K, Iwamoto N, Ono T (2011). Risk stratification for cardiac death in hemodialysis patients without obstructive coronary artery disease. Kidney Int.

[9] Simental-Mendía LE, Rodríguez-Morán M, Guerrero-Romero F (2008). The product of fasting glucose and triglycerides as surrogate for identifying insulin resistance in apparently healthy subjects. Metab Syndr Relat Disord.

[10] Gastaldelli A (2022). Measuring and estimating insulin resistance in clinical and research settings. Obesity (Silver Spring).

[11] Mirjalili SR, Soltani S, Heidari Meybodi Z, Marques-Vidal P, Kraemer A, Sarebanhassanabadi M (2023). An innovative model for predicting coronary heart disease using triglyceride-glucose index: a machine learning-based cohort study. Cardiovasc Diabetol.

[12] Su J, Li Z, Huang M, Wang Y, Yang T, Ma M (2022). Triglyceride glucose index for the detection of the severity of coronary artery disease in different glucose metabolic states in patients with coronary heart disease: a RCSCD-TCM study in China. Cardiovasc Diabetol.

[13] Park K, Ahn CW, Lee SB, Kang S, Nam JS, Lee BK (2019). Elevated TyG index predicts progression of coronary artery calcification. Diabetes Care.

[14] Wu Z, Guo D, Chen S, Sun X, Zhang Y, Liu X (2023). Combination of the triglyceride-glucose index and EuroSCORE II improves the prediction of long-term adverse outcomes in patients undergoing coronary artery bypass grafting. Diabetes Metab Res Rev.

[15] Baek J, He C, Afshinnia F, Michailidis G, Pennathur S (2022). Lipidomic approaches to dissect dysregulated lipid metabolism in kidney disease. Nat Rev Nephrol.

[16] Legouis D, Faivre A, Cippà PE, de Seigneux S (2022). Renal gluconeogenesis: an underestimated role of the kidney in systemic glucose metabolism. Nephrol Dial Transplant.

[17] Kilpatrick RD, McAllister CJ, Kovesdy CP, Derose SF, Kopple JD, Kalantar-Zadeh K (2007). Association between serum lipids and survival in hemodialysis patients and impact of race. J Am Soc Nephrol.

[18] Soohoo M, Moradi H, Obi Y, Kovesdy CP, Kalantar-Zadeh K, Streja E (2019). Serum triglycerides and mortality risk across stages of chronic kidney disease in 2 million U.S. veterans. J Clin Lipidol.

[19] Reiss AB, Voloshyna I, De Leon J, Miyawaki N, Mattana J (2015). Cholesterol Metabolism in CKD. Am J Kidney Dis.

[20] Abe M, Kalantar-Zadeh K (2015). Haemodialysis-induced hypoglycaemia and glycaemic disarrays. Nat Rev Nephrol.

[21] Hill CJ, Maxwell AP, Cardwell CR, Freedman BI, Tonelli M, Emoto M (2014). Glycated hemoglobin and risk of death in diabetic patients treated with hemodialysis: a meta-analysis. Am J Kidney Dis.

[22] Granger CB, Goldberg RJ, Dabbous O, Pieper KS, Eagle KA, Cannon CP (2003). Predictors of hospital mortality in the global registry of acute coronary events. Arch Intern Med.

[23] Alizargar J, Bai C-H, Hsieh N-C, Wu S-FV. Use of the triglyceride-glucose index (TyG) in cardiovascular disease patients. Cardiovasc Diabetol 2020;19:8. 10.1186/s12933-019-0982-2 PMC6963998 31941513

[24] Wu Z, Liu L, Wang W, Cui H, Zhang Y, Xu J (2022). Triglyceride-glucose index in the prediction of adverse cardiovascular events in patients with premature coronary artery disease: a retrospective cohort study. Cardiovasc Diabetol.

[25] Jin J-L, Cao Y-X, Wu L-G, You X-D, Guo Y-L, Wu N-Q (2018). Triglyceride glucose index for predicting cardiovascular outcomes in patients with coronary artery disease. J Thorac Dis.

[26] Ye Z, An S, Gao Y, Xie E, Zhao X, Guo Z (2023). Association between the triglyceride glucose index and in-hospital and 1-year mortality in patients with chronic kidney disease and coronary artery disease in the intensive care unit. Cardiovasc Diabetol.

[27] Liyanage T, Ninomiya T, Jha V, Neal B, Patrice HM, Okpechi I (2015). Worldwide access to treatment for end-stage kidney disease: a systematic review. Lancet.

[28] Xiong S, Chen Q, Zhang Z, Chen Y, Hou J, Cui C (2022). A synergistic effect of the triglyceride-glucose index and the residual SYNTAX score on the prediction of intermediate-term major adverse cardiac events in patients with type 2 diabetes mellitus undergoing percutaneous coronary intervention. Cardiovasc Diabetol.

[29] Radcliffe NJ, Seah J-M, Clarke M, MacIsaac RJ, Jerums G, Ekinci EI (2017). Clinical predictive factors in diabetic kidney disease progression. J Diabetes Investig.

[30] Dave N, Wu J, Thomas S (2018). Chronic kidney disease-induced insulin resistance: current state of the field. Curr Diab Rep.

[31] Nakashima A, Kato K, Ohkido I, Yokoo T (2021). Role and treatment of insulin resistance in patients with chronic kidney disease: a review. Nutrients.

[32] Nishikawa T, Kukidome D, Sonoda K, Fujisawa K, Matsuhisa T, Motoshima H (2007). Impact of mitochondrial ROS production in the pathogenesis of insulin resistance. Diabetes Res Clin Pract.

[33] Yang Q, Vijayakumar A, Kahn BB (2018). Metabolites as regulators of insulin sensitivity and metabolism. Nat Rev Mol Cell Biol.

[34] Tao L-C, Xu J, Wang T, Hua F, Li J-J (2022). Triglyceride-glucose index as a marker in cardiovascular diseases: landscape and limitations. Cardiovasc Diabetol.

[35] Samuel VT, Shulman GI (2016). The pathogenesis of insulin resistance: integrating signaling pathways and substrate flux. J Clin Invest.

[36] Jia G, DeMarco VG, Sowers JR (2016). Insulin resistance and hyperinsulinaemia in diabetic cardiomyopathy. Nat Rev Endocrinol.

[37] Cho Y-R, Ann SH, Won K-B, Park G-M, Kim Y-G, Yang DH (2019). Association between insulin resistance, hyperglycemia, and coronary artery disease according to the presence of diabetes. Sci Rep.

[38] Zhang R, Shi S, Chen W, Wang Y, Lin X, Zhao Y (2023). Independent effects of the triglyceride-glucose index on all-cause mortality in critically ill patients with coronary heart disease: analysis of the MIMIC-III database. Cardiovasc Diabetol.

[39] Zhang Y, Ding X, Hua B, Liu Q, Gao H, Chen H (2020). High triglyceride-glucose index is associated with adverse cardiovascular outcomes in patients with acute myocardial infarction. Nutr Metab Cardiovasc Dis.

[40] Zou S, Xu Y (2021). Association of the triglyceride-glucose index and major adverse cardiac and cerebrovascular events in female patients undergoing percutaneous coronary intervention with drug-eluting stents: A retrospective study. Diabetes Res Clin Pract.

[41] Lawton JS, Tamis-Holland JE, Bangalore S, Bates ER, Beckie TM, Bischoff JM (2022). 2021 ACC/AHA/SCAI Guideline for Coronary Artery Revascularization: A Report of the American College of Cardiology/American Heart Association Joint Committee on Clinical Practice Guidelines. J Am Coll Cardiol.

[42] Collet J-P, Thiele H, Barbato E, Barthélémy O, Bauersachs J, Bhatt DL (2021). 2020 ESC Guidelines for the management of acute coronary syndromes in patients presenting without persistent ST-segment elevation. Eur Heart J.

[43] Gurm HS, Gore JM, Anderson FAJ, Wyman A, Fox KAA, Steg PG (2012). Comparison of acute coronary syndrome in patients receiving versus not receiving chronic dialysis (from the Global Registry of Acute Coronary Events [GRACE] Registry). Am J Cardiol.

[44] Xiong S, Chen Q, Chen X, Hou J, Chen Y, Long Y (2022). Adjustment of the GRACE score by the triglyceride glucose index improves the prediction of clinical outcomes in patients with acute coronary syndrome undergoing percutaneous coronary intervention. Cardiovasc Diabetol.

