# Supplementary material for: The triglyceride-glucose index predicts 1-year major adverse cardiovascular events in end-stage renal disease patients with coronary artery disease
Source: Cardiovasc Diabetol. 2023 Oct 27;22:292. doi: 10.1186/s12933-023-02028-7 (PMC10612201; doi:10.1186/s12933-023-02028-7)
Supplement: Supplementary file 1 — Additional file 1: Table S1. Cox regression models for the association of triglyceride-glucose index and secondary outcomes. Table S2. Baseline risk model for major adverse cardiovascular events. Table S3. Cox regression models for the association of triglyceride-glucose index and MACE after excluding patients who experienced a MACE event during hospitalization. Table S4. Cox regression models for the association of triglyceride-glucose index and MACE after excluding patients with blood glucose levels below 3.9 mmol/L. Table S5. Baseline clinical characteristics of patients before and after propensity score matching. Figure S1. Kaplan-Meier Estimated Event Rates of MACE according to tertiles of the TyG index after excluding patients who experienced a MACE event during hospitalization. Figure S2. Restricted cubic spline curves of the association between TyG index and MACE after excluding patients who experienced a MACE event during hospitalization. [file 12933_2023_2028_MOESM1_ESM.pdf]

Additional file 1

Table S1. Cox regression models for the association of triglyceride-glucose index and secondary outcomes

	**Univariate**			**Multivariable**		
	**HR**	**95%CI**	***P* value**	**HR**	**95%CI**	***P* value**
**All-cause death**						
Tertile 1	Ref.			Ref.		
Tertile 2	1.51	1.00-2.27	0.052	1.55	1.01-2.38	0.044
Tertile 3	1.71	1.14-2.56	0.009	1.62	1.06-2.48	0.026
**Non-fatal myocardial infarction**						
Tertile 1	Ref.			Ref.		
Tertile 2	1.27	0.61-2.65	0.517	1.20	0.57-2.50	0.634
Tertile 3	2.01	1.03-3.95	0.042	1.79	0.91-3.52	0.093
**Non-fatal stroke**						
Tertile 1	Ref.			Ref.		
Tertile 2	3.59	0.75-17.3	0.111	3.06	0.63-14.98	0.167
Tertile 3	1.59	0.27-9.51	0.612	1.07	0.17-6.58	0.945
**Gastrointestinal bleeding**						
Tertile 1	Ref.			Ref.		
Tertile 2	0.64	0.28-1.49	0.305	0.62	0.27-1.45	0.273
Tertile 3	1.11	0.54-2.31	0.771	0.99	0.48-2.06	0.978

CI, confidence interval; HR, hazard ratio; TyG, triglyceride-glucose.

Table S2. Baseline risk model for major adverse cardiovascular events

Variable	HR	95%CI	*P* value
Age	1.02	1.00-1.03	0.049
Male	0.95	0.69-1.31	0.772
Systolic blood pressure	0.99	0.98-1.00	0.124
Diastolic blood pressure	0.98	0.97-1.00	0.027
Heart rate	1.01	1.00-1.02	0.010
Diabetes mellitus	1.26	0.89-1.79	0.200
Atrial fibrillation	1.39	0.91-2.13	0.133
Cerebrovascular disease	1.54	1.11-2.13	0.009
Cause of end-stage renal disease			
Diabetes mellitus	Ref.		
Hypertension	1.28	0.79-2.06	0.315
Glomerulonephritis	0.78	0.50-1.22	0.285
Other/unknown	1.08	0.72-1.60	0.721
AMI as Index presentation	1.41	1.04-1.91	0.027
Any left main disease	1.77	1.25-2.50	0.001
3-vessel disease	1.86	1.36-2.53	<0.001
ACE inhibitor or ARB	0.84	0.63-1.12	0.223
Calcium-channel blocker	0.85	0.63-1.14	0.273

ACE, angiotensin-converting enzyme; AMI, acute myocardial infarction; ARB, angiotensin receptor blocker; CI, confidence interval; HR, hazard ratio.

Table S3. Cox regression models for the association of triglyceride-glucose index and MACE after excluding patients who experienced a MACE event during hospitalization

	**Model 1**			**Model 2**			**Model 3**		
	**HR**	**95%CI**	***P* value**	**HR**	**95%CI**	***P* value**	**HR**	**95%CI**	***P* value**
TyG index	1.39	1.14-1.70	<0.001	1.40	1.15-1.70	<0.001	1.34	1.10-1.63	0.004
Tertile 1	Ref.			Ref.			Ref.		
Tertile 2	1.47	1.00-2.16	0.052	1.53	1.04-2.25	0.032	1.49	1.01-2.20	0.047
Tertile 3	1.66	1.14-2.43	0.009	1.66	1.14-2.43	0.009	1.55	1.06-2.28	0.024

Model 1: unadjusted. Model 2: adjusted for age and sex. Model 3: adjusted for age, sex, systolic blood pressure, diastolic blood pressure, acute myocardial infarction as index presentation, left main disease, 3-vessel disease, angiotensin-converting enzyme inhibitor or angiotensin receptor blocker, and calcium-channel blocker.

CI, confidence interval; HR, hazard ratio; MACE, major adverse cardiovascular events; TyG, triglyceride-glucose.

Table S4. Cox regression models for the association of triglyceride-glucose index and MACE after excluding patients with blood glucose levels below 3.9 mmol/L

	**Model 1**			**Model 2**			**Model 3**		
	**HR**	**95%CI**	***P* value**	**HR**	**95%CI**	***P* value**	**HR**	**95%CI**	***P* value**
TyG index	1.41	1.17-1.70	<0.001	1.43	1.18-1.72	<0.001	1.34	1.09-1.64	0.005
Tertile 1	Ref.			Ref.			Ref.		
Tertile 2	1.43	0.99-2.06	0.059	1.52	1.05-2.20	0.027	1.48	1.01-2.16	0.046
Tertile 3	1.73	1.21-2.47	0.003	1.76	1.23-2.51	0.002	1.62	1.11-2.36	0.011

Model 1: unadjusted. Model 2: adjusted for age and sex. Model 3: adjusted for age, sex, systolic blood pressure, diastolic blood pressure, heart rate, diabetes mellitus, atrial fibrillation, cause of dialysis, acute myocardial infarction as index presentation, left main disease, 3-vessel disease, angiotensin-converting enzyme inhibitor or angiotensin receptor blocker, and calcium-channel blocker.

CI, confidence interval; HR, hazard ratio; MACE, major adverse cardiovascular events; TyG, triglyceride-glucose.

Table S5. Baseline clinical characteristics of patients before and after propensity score matching

	Before matching			After matching		
**Characteristic**	**≤ 8.99 (n=423)**	**>8.99 (n=536)**	**Std Diff**	**≤ 8.99 (n=265)**	**>8.99 (n=265)**	**Std Diff**
Age, mean (SD), yrs	61.6 (10.9)	62.0 (10.2)	0.040	61.9 (10.3)	62.4 (9.9)	0.054
Male, No. (%)	325 (76.8)	390 (72.8)	0.094	199 (75.1)	207 (78.1)	0.071
SBP, mean (SD), mmHg	142.9 (24.6)	140.6 (25.2)	0.093	140.7 (24.1)	141.8 (25.5)	0.046
DBP, mean (SD), mmHg	79.6 (13.4)	78.0 (13.4)	0.120	78.5 (13.2)	78.3 (14.2)	0.018
Heart rate, mean (SD), beats/min	79.3 (14.7)	81.4 (14.6)	0.143	80.4 (15.1)	79.7 (13.5)	0.044
Medical history and risk factors, No. (%)						
Hypertension	384 (90.8)	506 (94.4)	0.139	241 (90.9)	247 (93.2)	0.084
Diabetes mellitus	163 (38.5)	346 (64.6)	0.539	130 (49.1)	134 (50.6)	0.030
Current smoker	92 (21.7)	89 (16.6)	0.131	48 (18.1)	51 (19.2)	0.029
Atrial fibrillation	37 (8.7)	44 (8.2)	0.019	25 (9.4)	28 (10.6)	0.038
Cerebrovascular disease	77 (18.2)	107 (20.0)	0.045	48 (18.1)	47 (17.7)	0.010
Valvular disease	16 (3.8)	15 (2.8)	0.055	7 (2.6)	8 (3.0)	0.023
Peripheral arterial disease	36 (8.5)	59 (11.0)	0.084	25 (9.4)	23 (8.7)	0.026
Cause of ESRD, No. (%)			0.417			0.036
Diabetes mellitus	94 (22.2)	210 (39.2)		80 (30.2)	82 (30.9)	
Hypertension	67 (15.8)	56 (10.4)		35 (13.2)	35 (13.2)	
Glomerulonephritis	128 (30.3)	102 (19.0)		66 (24.9)	62 (23.4)	
Other/unknown	134 (31.7)	168 (31.3)		84 (31.7)	86 (32.5)	
Dialysis modality, No. (%)			0.160			0.016
Hemodialysis	401 (94.8)	486 (90.7)		249 (94.0)	248 (93.6)	
Peritoneal dialysis	22 (5.2)	50 (9.3)		16 (6.0)	17 (6.4)	
Vintage, yrs			0.130			0.020
<1	87 (20.6)	112 (20.9)		55 (20.8)	56 (21.1)	

1-5	183 (43.3)	261 (48.7)		120 (45.3)	118 (44.5)	
5-10	119 (28.1)	129 (24.1)		71 (26.8)	71 (26.8)	
≥10	34 (8.0)	34 (6.3)		19 (7.2)	20 (7.5)	
Index presentation, No. (%)			0.162			<0.001
AMI	230 (54.4)	334 (62.3)		155 (58.5)	155 (58.5)	
Non-AMI	193 (45.6)	202 (37.7)		110 (41.5)	110 (41.5)	
Hemoglobin, g/L	105.6 (19.4)	105.4 (20.1)	0.009	105.0 (19.4)	105.3 (20.3)	0.015
Glucose, mmol/L	5.2 (1.5)	9.6 (4.6)	1.275	5.3 (1.6)	9.2 (4.3)	1.211
Serum creatinine, mg/dl	9.0 (3.0)	8.8 (3.4)	0.078	8.7 (2.8)	8.9 (3.5)	0.046
TG, mmol/L	1.2 (0.5)	2.3 (1.0)	1.471	1.2 (0.5)	2.1 (0.9)	1.255
TC, mmol/L	3.6 (0.9)	4.0 (1.2)	0.361	3.8 (1.0)	3.8 (1.2)	0.051
HDL-C, mmol/L	1.0 (0.3)	0.9 (0.3)	0.407	1.0 (0.3)	1.0 (0.4)	0.070
LDL-C, mmol/L	2.2 (0.8)	2.4 (0.9)	0.223	2.2 (0.9)	2.2 (0.9)	0.005
TyG index	8.4 (0.5)	9.6 (0.5)	2.584	8.5 (0.4)	9.5 (0.4)	2.514
Procedure characteristic, No. (%)						
Radial access	324 (76.6)	416 (77.6)	0.024	201 (75.8)	206 (77.7)	0.045
Extent of disease						
Any left main disease	45 (10.6)	66 (12.3)	0.053	30 (11.3)	28 (10.6)	0.024
2-vessel disease	113 (26.7)	150 (28.0)	0.029	78 (29.4)	77 (29.1)	0.008
3-vessel disease	220 (52.0)	324 (60.4)	0.171	153 (57.7)	150 (56.6)	0.023
Moderate or severe calcification	183 (43.3)	249 (46.5)	0.064	122 (46.0)	119 (44.9)	0.023
PCI treatment	292 (69.0)	384 (71.6)	0.057	181 (68.3)	187 (70.6)	0.049
Discharge medications, No. (%)						
Dual antiplatelet therapy	372 (87.9)	464 (86.6)	0.041	233 (87.9)	231 (87.2)	0.023
ACE inhibitor or ARB	204 (48.2)	237 (44.2)	0.081	122 (46.0)	113 (42.6)	0.068
Beta-blocker	329 (77.8)	446 (83.2)	0.137	209 (78.9)	211 (79.6)	0.019

Calcium-channel blocker	290 (68.6)	330 (61.6)	0.147	164 (61.9)	165 (62.3)	0.008
Statin	393 (92.9)	506 (94.4)	0.061	251 (94.7)	249 (94.0)	0.033

Data are presented as mean (SD) or n (%).

ACE, angiotensin-converting enzyme; AMI, acute myocardial infarction; ARB, angiotensin receptor blocker; DBP, diastolic blood pressure; ESRD, end-stage renal disease; HDL-C, high-density lipoprotein cholesterol; LDL-C, low-density lipoprotein cholesterol; PCI, percutaneous coronary intervention; SBP, systolic blood pressure; TC, total cholesterol; TG, triglycerides; TyG, triglyceride-glucose.

Figure S1. Kaplan-Meier Estimated Event Rates of MACE according to tertiles of theTyG index after excluding patients who experienced a MACE event during hospitalization

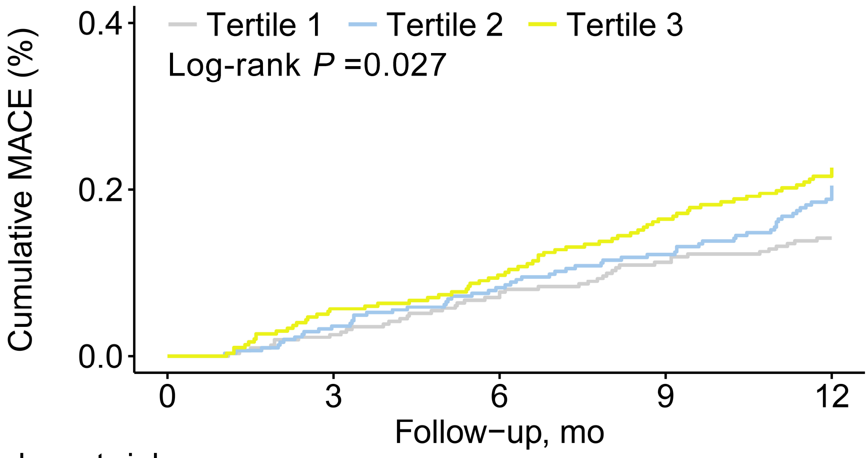

Number at risk

Tertile 1	312	304	290	274	264
Tertile 2	306	294	279	265	244
Tertile 3	300	281	268	247	231

MACE, major adverse cardiovascular events; TyG, triglyceride-glucose index.

Figure S2. Restricted cubic spline curves of the association between TyG index and MACE after excluding patients who experienced a MACE event during hospitalization.

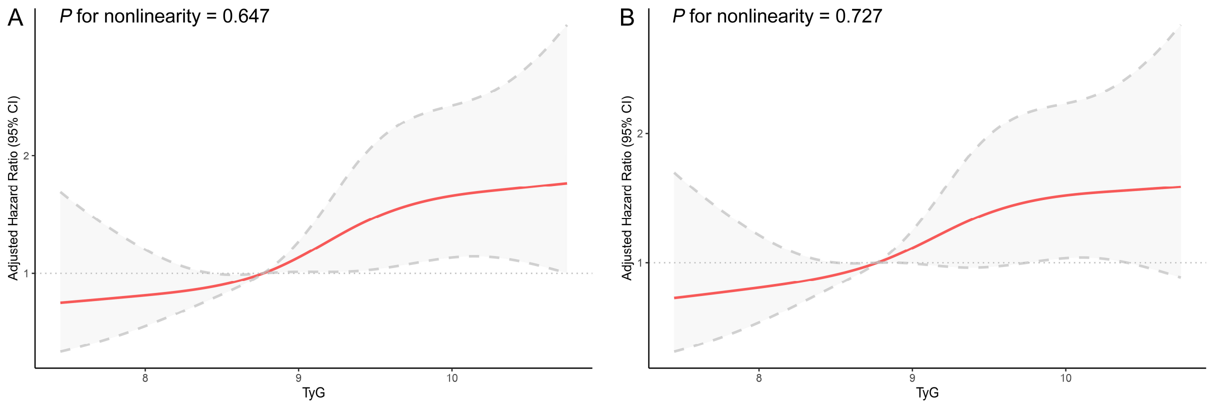

(A) unadjusted model. **(B)** fully adjusted model.

Hazard ratios are indicated by solid red lines and 95% CIs are indicated by shaded areas.

CI, confidence interval; MACE, major adverse cardiovascular events; TyG, triglyceride-glucose index.
